# Chemical Attributes of UK-Grown Tea and Identifying Catechin and Metabolite Dynamics in Green and Black Tea Using Metabolomics and Machine Learning

**DOI:** 10.3390/metabo16010084

**Published:** 2026-01-21

**Authors:** Amanda J. Lloyd, Jasen Finch, Alina Warren-Walker, Alison Watson, Laura Lyons, MJ Pilar Martinez Martin, Thomas Wilson, Manfred Beckmann

**Affiliations:** Department of Life Sciences, Aberystwyth University, Aberystwyth SY23 3FL, UKarw21@aber.ac.uk (A.W.-W.); aan@aber.ac.uk (A.W.); mam167@aber.ac.uk (M.P.M.M.); tpw2@aber.ac.uk (T.W.); meb@aber.ac.uk (M.B.)

**Keywords:** *Camellia sinensis*, flow infusion electrospray ionisation mass spectrometry (FIE-MS)

## Abstract

The Dartmoor Estate Tea plantation in Devon, UK, benefits from a unique microclimate and diverse soil conditions, which, together with its different processing methods, contribute to the distinctive flavours and chemical profiles of its teas. Objectives: The chemical diversity of Dartmoor tea was assessed via samples collected during processing of green and black tea. Methods: Leaf samples were collected during the processing of green and black tea and analysed using Flow Infusion Electrospray Ionisation Mass Spectrometry (FIE-MS). Results: For green tea processing, random forest regression identified features associated with the processing steps, resulting in a total of 272 *m*/*z* explanatory features. The analysis of black tea processing (4 h and overnight oxidation prior to roasting) yielded 209 discriminatory *m*/*z* features (4 h) and the model for the overnight oxidation and roasting treatments yielded 605 discriminatory *m*/*z* features. *K*-means clustering was performed on the percentage of relative abundance of the discriminatory *m*/*z* features. This grouped the discriminatory *m*/*z* features into 15 clusters of features showing similar trends across the processing stages. Functional and structural enrichment analysis was performed on each of the clusters and significant metabolic pathways included metabolism and biosynthesis of flavonoids, amino acids and lipids, the Pentose phosphate pathway, and the TCA cycle. Many discriminatory features were putatively classified as catechin-derived flavan-3-ols and flavonol glycosides. Conclusions: This research highlights the complex role that processing plays in shaping tea quality. It provides valuable insights into the metabolic pathways that influence tea production and emphasises how these factors determine the final chemical profile and sensory characteristics of tea.

## 1. Introduction

Tea is widely consumed in the UK and is made from the plant *Camellia sinensis* L. Regular tea consumption is associated with improved wellbeing, particularly healthy ageing, and a reduced risk of cardiovascular disease, certain cancers, liver disorders, obesity, and type 2 diabetes [1,2]. These benefits are largely attributed to the diverse bioactive compounds present in tea, which have been the subject of extensive research.

Tea contains an array of secondary metabolites, including polyphenols, which contribute significantly to its health-promoting properties. These metabolites, however, are influenced by several factors, such as plucking season, shading conditions, growth altitude, soil composition, climate, and the variety or cultivar of the tea plant, as well as the processing methods used [3,4]. These factors influence not only the chemical composition of tea but also its sensory qualities, including flavour, aroma, and colour, highlighting the complexity of tea as a functional food.

The health-promoting chemistry of tea includes catechins ((-)-epicatechin (EC), (-)-epigallocatechin (EGC), (-)-epicatechin-3-gallate (ECG), and (-)-epigallocatechin-3-gallate (EGCG)), theaflavins, thearubigins (tannins), and amino acids such as theanine [5]. These compounds contribute to the antioxidative, anti-inflammatory, and metabolic regulatory effects of tea. The processing of freshly plucked tea leaves into black (fermented), oolong (semi-fermented), green, and white (unfermented) teas further alters these bioactive compounds. Fermentation, for instance, involves enzymatic oxidation of catechins by endogenous polyphenol oxidases and peroxidases, leading to the formation of theaflavins and thearubigins. Previous work has shown that this process results in substantial catechin losses and transient peaks in theaflavins during black tea processing [6]. This process is accelerated by mechanical rolling, which ruptures the withered tea leaves, allowing the enzymes to react more efficiently with oxygen. In contrast, unfermented teas prevent enzymatic oxidation by inactivating these enzymes through steaming or pan-frying before rolling and drying [7,8].

The stages of tea processing, including withering, fixing/steaming (green tea), rolling, resting (a brief non-enzymatic relaxation period after rolling), and oxidation and drying (roasting), significantly influence the chemical composition and, consequently, the health benefits of the final product. Variations in processing methods affect the levels of key chemical constituents, such as polyphenols, as well as the sensory and functional attributes of tea [9,10,11].

Metabolomics has become a key tool for assessing the quality and authenticity of tea products. This advanced analytical approach enables unbiased screening of chemical compounds, allowing researchers to detect differences between tea samples based on their origin, processing methods, and storage conditions. Research has shown that metabolomics is highly effective for distinguishing tea varieties and identifying key quality markers [11,12,13,14]. Coupling metabolomics with traditional sensory evaluation and modern analytical techniques offers a complete picture of tea’s complexity and its potential health benefits.

Tea is both a part of cultural heritage and a functional beverage with many health benefits. Ongoing research into its bioactive compounds continues to reveal its diverse advantages, reinforcing its importance in both everyday diets and therapeutic contexts. The objective of this study was to characterise the chemical transformations occurring during green and black tea processing, with a particular focus on catechin dynamics and associated metabolic pathways, using an integrated untargeted metabolomics and machine learning approach applied to UK-grown tea.

## 2. Materials and Methods

### 2.1. Sample Collection

Fresh tea was picked from Dartmoor Estate Tea from two gardens on a morning in September 2023 where the air humidity was 58–65%, pressure 1016 hPa, and temperature 22 °C. Two leaves and a bud were selected and picked over a 3 h period, yielding over 12 kg of fresh leaf material. All harvested leaf material was thoroughly mixed and then divided into two batches intended for green tea processing and black tea processing, respectively. Throughout this section, “leaf” refers to the freshly plucked tea leaf, while “processed tea” refers to samples collected at specific stages of green or black tea manufacture.

For green tea, the leaf underwent a 4 h withering step (to approximately 90% moisture), followed by fixation in a wok (350 g batches, 5 min at 230 °C) to deactivate oxidative enzymes. The fixed leaf was then rolled for 1 h, allowed to rest briefly (non-enzymatic oxidation), and subsequently roasted at 75–115 °C for 3 h.

For black tea, withering samples were collected at three defined moisture levels—85% (9 h), 70% (23 h), and 60% (30 h). After withering, the leaf was rolled for 1 h and then divided into two oxidation treatments: a 4 h oxidation and an overnight (12 h) oxidation. Both oxidation treatments were followed by roasting at 75–115 °C for 3 h.

At each processing stage, leaf or processed tea samples were collected and immediately frozen in liquid nitrogen in 25 mL tubes for both green tea (picking; withering 90%; fixation; rolling; oxidation; roasting) and black tea (picking; withering 85%, 70%, 60%; rolling; 4 h or overnight oxidation; roasting). All samples were transported on dry ice and stored at −80 °C until analysis.

### 2.2. Chemicals and Reagents

Methanol, acetonitrile and formic acid of LC–MS grade were purchased from Fisher Scientific (Loughborough, UK). Ultrapure water was produced using a Milli-Q purification system (Millipore, Burlington, MA, USA). Four catechin standards—epicatechin (EC), epigallocatechin (EGC), epicatechin gallate (ECG), and epigallocatechin gallate (EGCG)—were purchased from Merck. Bligh and Dyer extraction reagents (chloroform, methanol, and water) were LC–MS or analytical grade. All chemicals were handled and stored according to the manufacturers’ instructions.

### 2.3. Sample Preparation and Extraction

The material collected at each processing stage for black and green tea varied in both size and moisture content. Therefore, a method was used to adjust all samples for weight and moisture content to allow direct comparison in the subsequent analyses. All procedures were carried out on ice. Prior to milling, one of the four replicates of each sample was weighed, freeze-dried and re-weighed, to determine the moisture content for this sample type. Mixer mill sample holders were pre-chilled at −80° and Bligh and Dyer extraction mix (chloroform/methanol/water 2:5:2) was prepared in advance and chilled to −20 °C, then stored on ice. The remaining three replicates were milled using a Retsch mixer mill with a stainless-steel ball. All sample tubes were first placed in liquid nitrogen before transferring to pre-chilled mixer mill holders, to prevent defrosting during milling. The samples were milled for 30 s at 30 Hz, then placed in liquid nitrogen once more. If necessary, the milling process was repeated until a visually homogeneous fine powder was obtained, with no visible leaf fragments larger than approximately 1 mm. A consistent amount (50 mg) of the milled material was spooned into pre-prepared weighed and labelled 2 mL Eppendorf tubes that contained 1 mL of chilled Bligh and Dyer extraction mix. The samples were then re-weighed to determine the exact amount of material added. All samples were vortexed, then shaken at 4 °C for 20 min before centrifugation at 13,000 rpm (~16,000× *g*) and 4 °C for 5 min. The supernatants were transferred into new labelled Eppendorf tubes and stored at −80 °C prior to analysis. Using both the moisture content and extraction weight data, adjustments were made to each sample, to normalise all extracts to the sample of lowest weight.

### 2.4. Sample Preparation for Analysis by Flow Infusion Electrospray Ionisation Mass Spectrometry (FIE-MS)

The tea leaf extracts were defrosted, vortexed, and spun down (13,000 rpm. (~16,000× *g*) and 4 °C for 5 min) before aliquoting. A preliminary trial had demonstrated that the optimum dilution for the samples was 1:10, so dilutions were prepared using Bligh and Dyer extraction mix before aliquoting 100 µL into HPLC vials with 200 µL inserts. For each FIE-MS sequence run, a quality control (QC) sample was prepared by combining 20 µL aliquots from all diluted samples in the run in a separate 5 mL Eppendorf tube and mixing before transferring 200 µL into an HPLC vial. Bligh and Dyer extraction mix (200 µL) was used as the control.

### 2.5. Flow Infusion Electrospray Ionisation Mass Spectrometry (FIE-MS)

FIE-MS was performed using an Exploris 120 mass analyser equipped with a Dionex Vanquish UHPLC system (Thermo Scientific, Waltham, MA, USA). Metabolite fingerprints were generated in both positive and negative ionisation modes, in a single run [15].

All samples were randomised to minimise batch effects. Samples (20 µL) were injected into a flow of 100 µL min^−1^ methanol/water (70:30, *v*/*v*). Ion intensities were acquired between *m*/*z* 55 and 1200 for 3.5 min at a resolution setting of 120,000, resulting in 3 (±1) ppm mass accuracy. Tuning and ESI source parameters were set according to manufacturer’s recommendations. Following data acquisition, Chromeleon.cmbx files were exported to .raw files and then converted to the .mzML open file format and centroided [16] using msconvert (TransProteomicPipeline 7.2.0) [17]. Spectral binning was applied using the R package binneR [18], and then standard post-acquisition processing routines were applied, including occupancy and QC filtering. Putative Molecular formulas were generated by using MZedDB V2.1 [19], an Aberystwyth University database for accurate mass annotation. The ionisation products of the assigned molecular formulas were initially searched against the KEGG compound database specific to *Camellia sinensis* for putative matches. Initial data analysis, including classification, was performed in R package metabolyseR.

### 2.6. Ultra-High-Performance Liquid Chromatography Coupled with Triple-Quadrupole Tandem Mass Spectrometry

To prepare the samples for HPLC-MS/MS analysis, once defrosted on ice they were vortexed to mix then spun down at 1250× *g* for 5 min at 4 °C. Each sample was diluted 50:50 using a solution of 70% acetonitrile plus 0.05% formic acid directly into 2 mL amber HPLC vials with inserts and crimp sealed. These were stored at 4 °C prior to analysis on the same day. Four standards were as follows: epicatechin (EC), epicatechin gallate (ECG), epigallocatechin (EGC) and epigallocatechin gallate (EGCG). Stock solutions (1 mg/mL) were prepared in 70% acetonitrile containing 0.05% formic acid. Sequential dilutions (1:10) were prepared to give standards at 0.01, 0.001, and 0.0001 mg/mL, which were used to construct standard curves for each catechin by HPLC-MS-MS analysis.

HPLC-MS/MS analysis was performed on a Thermo Scientific Vanquish UPLC system coupled with a TSQ Quantis triple-stage quadrupole mass spectrometer (Thermo Scientific), which was connected by an electrospray ionisation (ESI) source. The sheath gas was set at 50, the auxiliary gas at 10 and the sweep gas at 1 mL/min. The ion transfer tube temperature was 300 °C and the vaporizer temperature was set at 350 °C. MRM in positive ion mode was selected. Collision energies (eV) were optimised for each catechin standard, and the experiments were carried out monitoring the most intense transitions (Table 1 below).

Standards and samples were injected onto a Kinetex EVO C18 100A (2.1 mm × 150 mm, 1.7 µm) column (Phenomenex, Torrance, CA, USA). The flow rate was 0.25 mL/min and the column temperature was set at 30 °C. The sample volume was 5 µL. The HPLC conditions for the separation of catechins was based on the gradient method [20] For the separations, the mobile phase consisted of 0.1% aqueous formic acid (A) and methanol (B) in gradient elution at a flow rate of 0.25 mL/min. The optimised gradient elution was as follows: ramp from 0.0 to 25% B over 3 min and after a 3 min hold, the percentage of B was ramped to 30% over 10 min; then, at 17 min it was ramped to 100% B and held for 3 min before returning to 0% at 21 min, holding for 3 min. Using this chromatographic programme, the four catechins separated in the first 11 min and eluted in the order EGC, EC, EGCG, and ECG (Supplement S1). This methodology was used to confirm the catechins to Metabolomics Standards Initiative (MSI) Level 1.

### 2.7. Data Analysis

Data obtained was analysed using spectral binning [18] and further down-stream analysis. To provide a chemical overview of the samples, consensus structural classifications were compiled for each of the *m*/*z* features that were assigned a molecular formula, using the R package assignments which were developed as a ‘first pass’ automated assignment of molecular formulas to mass spectra acquired from FIE-MS (freely available from https://github.com/aberHRML/assignments, accessed 18 March 2024). The molecular formulas were initially searched against the KEGG compound database, and matching compounds were then filtered based on their ability to form the relevant adducts using MZedDB ionisation rules [19]. In MZedDB, the user can select appropriate rules manually from the adduct rules provided in the referenced MZedDB. For example, if the mobile phase does not contain NH_4_^+^ ions, then the rule {M + NH_4_^+^]^+^ is not appropriate and will not be included. Other automatic checks are applied, for example, if the molecular formula does not contain ‘N’, then the adduct rule [M + H-NH_3_]^+^ is not used. Where no compound matches were found in the KEGG compound database, the molecular formula was then searched in the PubChem compound database. Structural chemical classifications, based on the CHEMONT chemical taxonomy, were retrieved from the ClassyFire database for the matched compounds [21]. For each adduct linked to an assigned molecular formula, putative structural classifications were determined to a depth based on a consensus of 66% or higher among the matched compounds. FIE-MS are putative and fall under Metabolomics Standards Initiative (MSI) Level 3 (putatively characterised compound classes).

Random forest regression was used to identify *m*/*z* features related to processing steps. *K*-means clustering was performed on the percentage relative abundance of discriminatory *m*/*z* features. The number of clusters (k = 15) was selected based on a combination of the silhouette coefficient, inspection of within-cluster sum of squares (WCSS), and biological interpretability. Functional and structural enrichment analysis was carried out on each cluster to identify potential chemical classes and biological functions associated with cluster trends. Functional enrichment was performed using the PageRank approach implemented in the FELLA R package [22,23]. Structural enrichment was carried out on each cluster using over-representation analysis with Fisher’s exact test.

Welch’s *t*-test was performed on each of the *m*/*z* features to identify features that were significantly different between the 4 h and overnight oxidation treatments and between the roasted samples that had been subjected to 4 h and overnight oxidation. *p*-values were adjusted for multiple testing using the Benjamini–Hochberg false discovery rate (FDR) method.

## 3. Results

### 3.1. Results

**Figure 1:**
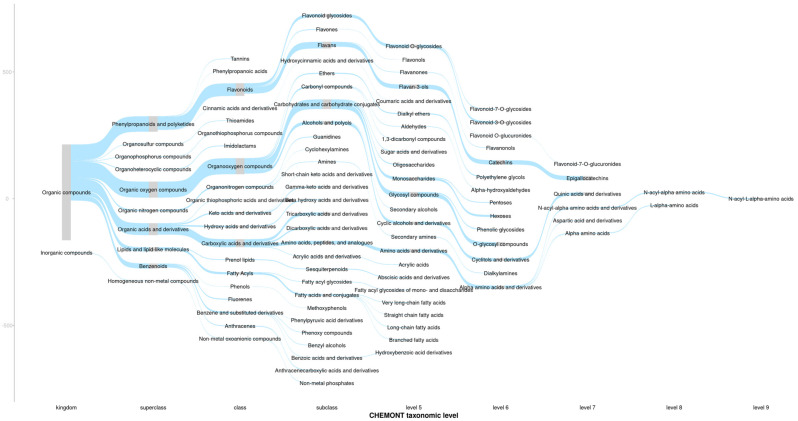
A Sankey plot providing an overview for the consensus structural classes of the assigned molecular formulas. Where grey bars show the frequencies of *m*/*z* features for each structural class.

The Sankey diagram (Figure 1) provides a compositional overview of the frequencies of the putative structural chemical classes assigned to the *m*/*z* features obtained from the samples. These frequencies are represented by the grey bars and blue flows within the diagram at each CHEMONT taxonomic level. There was a high frequency of *m*/*z* features putatively classified as phenylpropanoids and polyketides, as well as organic oxygen compounds and organic acids and their derivatives. These features indicated a high abundance of compounds commonly associated with tea quality and health benefits [24].

### 3.2. Green Tea

**Figure 2:**
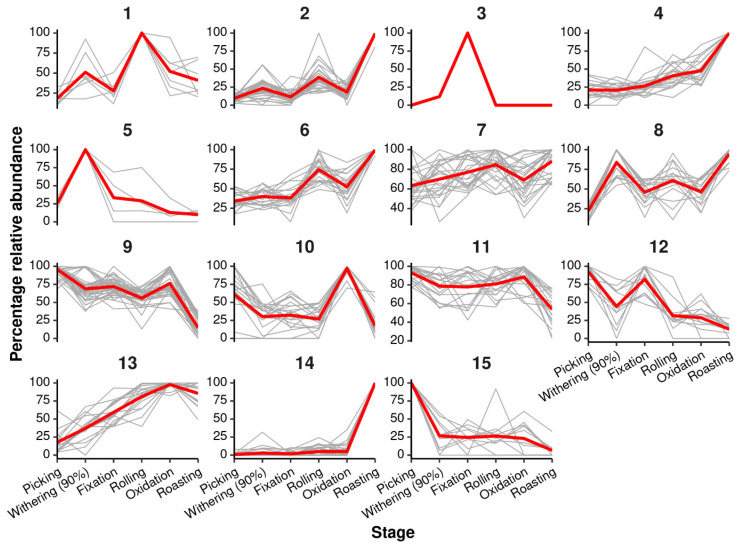
Clusters of discriminatory *m*/*z* features trends across processing steps of green tea. Where clusters were identified using k-means clustering and average clusters are shown in red. Withering to approximately 90% moisture.

Random forest regression was used to identify *m*/*z* features related to green tea processing steps. This yielded a strong model with a significant *R*^2^ value of 0.724 (*p* < 0.01, 3000 permutations). There were 272 *m*/*z* features that were found to be explanatory (% increase in mean squared error, *p* < 0.05, 3000 permutations). *K*-means clustering was performed on the percentage relative abundance of the discriminatory *m*/*z* features. This grouped the discriminatory *m*/*z* features into 15 clusters of features, showing similar trends across the processing steps. An overview of the clusters is shown in Figure 2. Significant putative metabolic pathways include Cluster 2 (Biosynthesis of amino acids), 4 (Flavonoid biosynthesis), 6 (Flavonoid and Carotenoid biosynthesis), 7 (Pentose phosphate pathway, Starch and sucrose metabolism, lipid metabolism), 8 (Starch and sucrose metabolism, lipid metabolism), 9 (TCA cycle, Lysine biosynthesis, C5-Branched dibasic acid metabolism), and 11 (TCA cycle, amino acid metabolism), as shown in full in Table 1. The prominence of flavonoid biosynthesis and carbohydrate metabolism pathways indicates that green tea processing preserves bioactive compounds beneficial for health (Table 1) [25]. Within the flavonoid-enriched clusters (e.g., Clusters 4 and 6), many discriminatory *m*/*z* features were putatively annotated (MSI level 3) as catechin-like flavan-3-ols and flavonol glycosides, whereas clusters associated with carbohydrate metabolism (e.g., Clusters 7 and 8) were dominated by features consistent with mono- and disaccharide-related metabolites. These representative metabolite classes help to contextualise the cluster trends across green tea processing.

### 3.3. Black Tea

Analysis of the black tea processing samples was more complex due to the presence of two branches of the process that included 4 h and overnight oxidation prior to roasting. To analyse this, random forest regression was performed by separately including the 4 h and overnight oxidation and roasting treatments with the prior process stages to identify chemistry of interest across the process in each case. The separate oxidation and roasting treatments were then compared directly using *t*-tests to identify the chemistry specifically related to these treatments.

### 3.4. Black Tea—4 h

**Figure 3:**
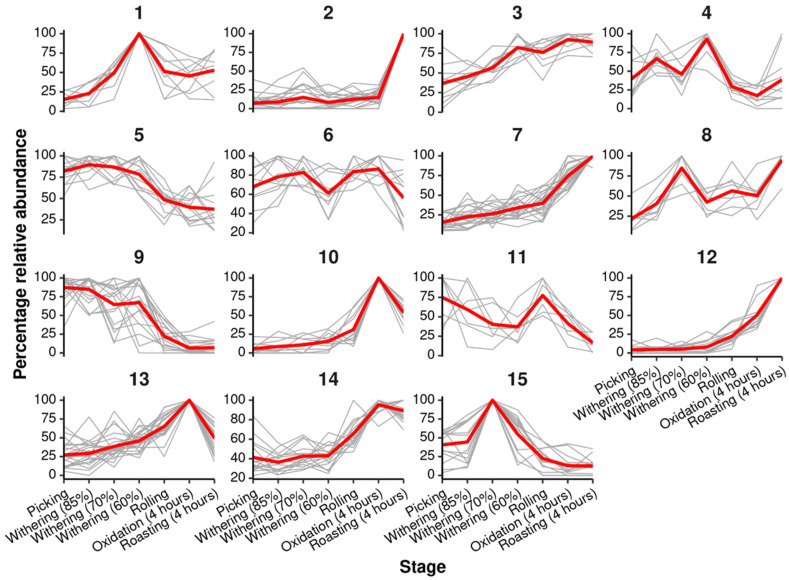
Clusters of discriminatory *m*/*z* features trends across processing steps of black tea (4 h oxidation). Where clusters were identified using k-means clustering and average clusters are shown in red. moisture levels 85% (9 h), 70% (23 h), and 60% (30 h). 4 h oxidation and an overnight (12 h) oxidation.

Random forest regression was performed using the process steps and the 4 h oxidation and roasting treatments. This yielded a very strong model with a significant *R*^2^ value of 0.92 (*p* < 0.01, 3000 permutations). There were 209 *m*/*z* features that were found to be discriminatory (% increase in mean squared error, *p* < 0.05, 3000 permutations). *k*-means clustering was performed on the percentage relative abundance of the discriminatory *m*/*z* features. This grouped the discriminatory *m*/*z* features into 15 clusters of features, showing similar trends across the processing stages. An overview of the clusters is shown Figure 3. Functional and structural enrichment analysis was performed on each of the clusters. Discriminatory *m*/*z* clusters include Cluster 3 (TCA cycle, Pentose phosphate pathway, Carbon metabolism, amino acid metabolism), 5 (C5-Branched dibasic acid metabolism, Ascorbate and aldarate metabolism), 7 (Tyrosine metabolism, Phenylpropanoid biosynthesis, Ubiquinone and other terpenoid-quinone biosynthesis), 8 (Flavonoid biosynthesis, Flavone and flavonol biosynthesis), 9 (Flavone and flavonol biosynthesis), 10 (Arginine biosynthesis, Glyoxylate and dicarboxylate metabolism), 12 (Fructose and mannose metabolism, Galactose metabolism, N-glycan biosynthesis, lipid metabolism, Protein processing), 13 (Pentose and glucuronate interconversions, amino acid metabolism, Nucleotide metabolism), 14 (amino acid metabolism, Biosynthesis of secondary metabolites, Butanoate metabolism, 2-Oxocarboxylic acid metabolism), and 15 (Flavonoid biosynthesis), as shown in full in Table 1. In the 4 h oxidation branch, clusters linked to phenylpropanoid and flavonoid biosynthesis (e.g., clusters 7–9 and 15) contained discriminatory features putatively annotated as phenolic acids, catechin-like flavan-3-ols and flavonol glycosides. By contrast, clusters enriched for saccharide and N-glycan pathways (e.g., cluster 12) were dominated by sugar- and glycan-related features. These representative metabolite classes illustrate how early oxidation and roasting reshape both phenolic and carbohydrate-related chemistry.

### 3.5. Black Tea—Overnight

**Figure 4:**
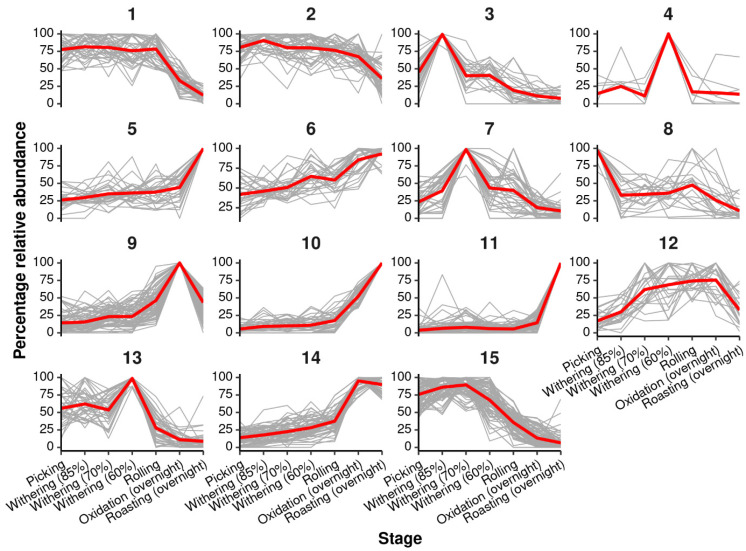
Clusters of discriminatory *m*/*z* feature trends across processing steps of black tea (overnight oxidation), where clusters were identified using k-means clustering and average clusters are shown in red. moisture levels 85% (9 h), 70% (23 h), and 60% (30 h). 4 h oxidation and an overnight (12 h) oxidation.

Random forest regression was performed using the process steps and the overnight oxidation and roasting treatments. This yielded a very strong model with a significant *R*^2^ value of 0.883 (*p* < 0.01, 3000 permutations). There were 605 *m*/*z* features that were found to be discriminatory (% increase in mean squared error, *p* < 0.05, 3000 permutations). *K*-means clustering was performed on the percentage relative abundance of the discriminatory *m*/*z* features. This grouped the discriminatory *m*/*z* features into 15 clusters of features, showing similar trends across the processing stages. An overview of the clusters is shown in Figure 4. Functional and structural enrichment analysis was performed on each of the clusters. Discriminatory *m*/*z* clusters include 1 (TCA cycle, Ascorbate and aldarate metabolism, C5-Branched dibasic acid metabolism), 2 (TCA cycle, amino acid metabolism/biosynthesis, C5-Branched dibasic acid metabolism), 3 (TCA cycle, Starch and sucrose metabolism, lipid metabolism, ABC transporters), 5 (Fructose and mannose metabolism, N-Glycan biosynthesis, Protein processing in endoplasmic reticulum), 6 (Pentose phosphate pathway, Fructose and mannose metabolism, amino acid degradation/biosynthesis, Amino sugar and nucleotide sugar metabolism), 7 (Flavonoid biosynthesis), 8 (Tyrosine metabolism), 9 (Pentose phosphate pathway, Pentose and glucuronate interconversions, amino acid biosynthesis, Tropane, piperidine and pyridine alkaloid), 11 (Biosynthesis of secondary metabolites), 12 (amino acid biosynthesis, Glyoxylate and dicarboxylate metabolism, Pantothenate and CoA biosynthesis), 13 (Biosynthesis of secondary metabolites), 14 (Pentose phosphate pathway, Vitamin B6 metabolism, amino acid biosynthesis/degradation/metabolism, Phenylpropanoid biosynthesis), and 15 (TCA cycle, amino acid biosynthesis, C5-Branched dibasic acid metabolism, 2-Oxocarboxylic acid metabolism), as shown in full in Table 1. The overnight oxidation treatment led to further biochemical transformations, particularly in flavonoid biosynthesis and the TCA cycle, with increased metabolic diversity observed in overnight oxidation compared to the 4 h treatment. For the overnight oxidation branch, flavonoid-enriched clusters (e.g., Cluster 7) were characterised by features putatively annotated as flavan-3-ols and their oxidised derivatives, whereas clusters associated with the TCA cycle and related energy metabolism (e.g., Clusters 1–3 and 15) contained small organic and dicarboxylic acid-like features. These representative examples support the view that extended oxidation drives both deeper polyphenol transformation and broader shifts in core carbon metabolism.

### 3.6. Oxidation and Roasting

Welch’s *t*-test was performed on each of the *m*/*z* features to identify features that were significantly different between the 4 h and overnight oxidation treatments, and between the roasted samples that had been subjected to 4 h and overnight oxidation. Between the 4 h and overnight oxidation treatments identified, a total of 307 features were significantly different. Between the roasted samples that had been subjected to 4 h and overnight oxidation, there was a total of 701 features that were significantly different. Functional enrichment analysis was performed separately on the *m*/*z* features that were found to be increased or decreased in each comparison. The significantly enriched metabolic pathways are shown in Table 2. Similarly, structural enrichment analysis was performed to identify the structural classes of interest amongst the features that were increased or decreased in the overnight oxidation treatment compared to the 4 h oxidation treatment and in the overnight treatment compared to the 4 h treatment. Flavonoids, amino acids, and lipids appeared to show changes, among other chemical reactions, between Oxidation (4 h)~Oxidation (overnight) and Roasting (4 h)~Roasting (overnight) (Table 2). Overnight oxidation resulted in a notable reduction in catechins and an increase in flavonoid derivatives, likely due to extended enzymatic oxidation [26].

### 3.7. Catechins in Black and Green Tea

**Figure 5:**
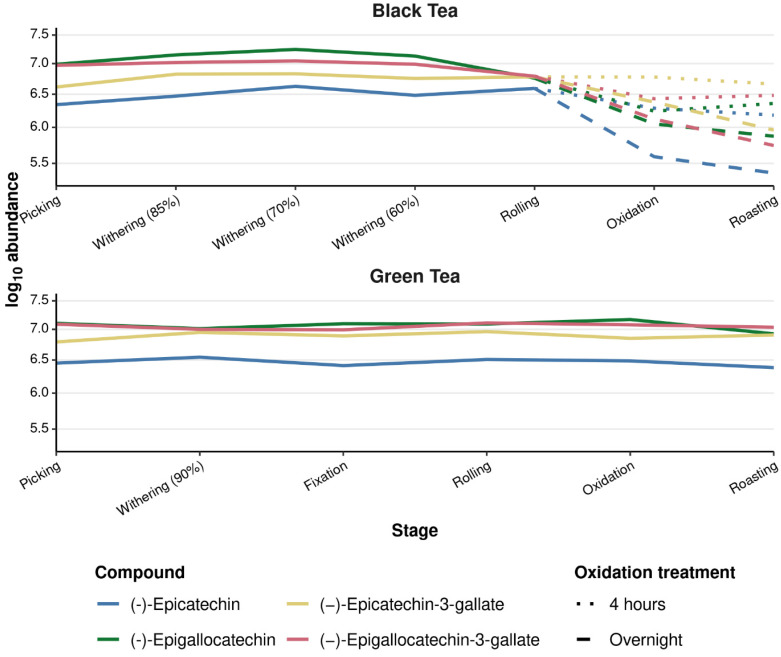
Using HPLC-MS/MS, the four catechins, EC, ECG, EGC, and EGCG, were confirmed and quantified in green and black tea over processing, where units = log_10_-transformed normalised peak area.

Using targeted HPLC-MS/MS, the four major catechins—(-)-epicatechin (EC), (-)-epigallocatechin (EGC), (-)-epicatechin-3-gallate (ECG) and (-)-epigallocatechin-3-gallate (EGCG)—were confirmed and quantified across all processing stages (Figure 5). In black tea, log_10_-transformed abundances at picking ranged from approximately 6.4 for EC to around 7.1–7.2 for EGC and EGCG, indicating that all four catechins were present at relatively high and comparable levels. During withering (85–60%), a slight increase of about 0.1–0.3 log_10_ units was observed for each catechin, consistent with a modest concentration effect and limited oxidative loss at this stage.

Subsequent processing produced clear differences between the 4 h and overnight oxidation treatments. After rolling, catechin levels began to decline, but in the 4 h oxidation branch, EC, EGC, ECG, and EGCG decreased only gradually, by roughly 0.2–0.3 log_10_ units over oxidation and roasting. In contrast, overnight oxidation caused a pronounced depletion of all four catechins. By the end of roasting, EC showed almost a 1 log_10_ unit decrease relative to the rolled leaf, while EGC and EGCG decreased by approximately 0.5–0.7 log_10_ units and ECG by about 0.4 log_10_ units. Because these values are log-transformed, the observed decreases correspond to several-fold reductions in catechin abundance, indicating extensive oxidative conversion of monomeric flavan-3-ols into higher-molecular-weight oxidation products such as theaflavins and thearubigins [6,26].

Green tea displayed a markedly different behaviour. Across picking, withering (90%), fixation, rolling, oxidation, and roasting, the abundances of all four catechins remained within a narrow range (generally <0.2 log_10_ units variation), with EGC and EGCG consistently the most abundant, followed by ECG and EC. These stable profiles show that green tea processing, which involves early enzyme inactivation during fixation, effectively preserves catechins and prevents the extensive oxidative degradation seen in black tea. Overall, the established concentration levels and processing-dependent trends for each catechin underline their central role in determining the biological potential of tea and support the view that stronger oxidation reduces catechin-derived bioactivity while contributing to the formation of characteristic black tea pigments [26].

## 4. Discussion

This study provided an exploration of the chemical diversity and transformation of tea during its processing stages, focusing on green and black tea from Dartmoor Estate Tea. The findings revealed important insights into the metabolic pathways and chemical changes associated with the processing steps. For green tea, metabolomic analysis putatively identified significant *m*/*z* features associated with the processing steps, which were enriched in pathways such as flavonoid biosynthesis, starch and sucrose metabolism, lipid metabolism, and the TCA cycle. These pathways are essential for maintaining the antioxidant properties and overall bioactive profile of green tea, which have been linked to various health benefits, including anti-inflammatory and cardioprotective effects [24,25].

The results indicate that green tea processing preserves catechins and other polyphenols crucial for its functional properties. The retention of these compounds aligns with previous studies demonstrating that non-fermented tea varieties maintain higher levels of antioxidants compared to black tea, where oxidation leads to significant chemical transformations [26]. Understanding these metabolic pathways provides a foundation for optimising tea processing methods to enhance the retention of health-promoting compounds, supporting consumer demand for high-quality, functional beverages.

The interplay between lipid metabolism and flavonoid biosynthesis suggests that tea processing affects not only the phenolic profile but also lipid-derived volatiles, which contribute to aroma and taste. These findings highlight the need for further research into the role of lipid transformations in shaping the sensory attributes of tea products, particularly in relation to consumer preferences and potential therapeutic applications.

Black tea demonstrated greater chemical complexity, with its polyphenolic profile strongly shaped by oxidation duration and subsequent roasting. HPLC-MS/MS analysis showed that while green tea retained its catechins throughout processing, black tea exhibited substantial catechin loss—most notably under overnight oxidation, whereas a 4 h oxidation caused only a slight decline. This pronounced reduction aligns with previous studies indicating that extended oxidation promotes the enzymatic and non-enzymatic conversion of catechins into theaflavins and thearubigins, compounds responsible for black tea’s colour, flavour, and characteristic astringency [6,7,8]. Studies on black tea manufacture have reported >90% losses of key catechins alongside transient peaks in theaflavins during fermentation, illustrating the scale of these oxidative transformations [6]. These oxidation-derived transformations not only define black tea’s sensory attributes and flavour complexity but also influence its antioxidant activity and overall functional properties, underscoring the need for precise control of oxidation conditions to optimise both quality and potential health benefits.

A more detailed inspection of the individual catechins supports these observations. The non-gallated catechins (EC and EGC) showed moderate declines under both oxidation treatments, whereas the gallated catechins (ECG and EGCG) exhibited much greater reductions—particularly under overnight oxidation. This reflects the higher oxidative susceptibility of gallated catechins, whose additional galloyl moieties enhance their reactivity with polyphenol oxidase and peroxidase, accelerating their conversion to theaflavin-3-gallate, theaflavin-3,3′-digallate, and subsequent thearubigin polymers. While theaflavins and thearubigins were not directly quantified, the observed patterns are consistent with established oxidative transformations during tea processing. The pronounced depletion of EGCG and EGC therefore highlights their central roles as primary substrates driving the formation of oxidation-derived pigments that shape black tea’s colour and briskness. In contrast, green tea showed stable levels of all four catechins across processing, confirming that early heat fixation effectively inactivates oxidative enzymes, thereby preserving flavan-3-ols that underpin green tea’s antioxidant potential. Together, these catechin-specific transformations provide deeper insight into how processing intensity modulates the sensory profile and biological functionality of tea, linking oxidation-driven chemistry to differences in health-promoting potential between black and green teas.

Functional enrichment analysis putatively revealed that pathways such as amino acid metabolism, flavonoid biosynthesis, and the pentose phosphate pathway played significant roles. These pathways play a critical role in shaping the chemical profile of black tea [24,25]. The pentose phosphate pathway, in particular, is linked to the generation of NADPH, which facilitates redox balance during oxidation and contributes to the biochemical stability of flavonoid compounds. Understanding these metabolic shifts provides valuable insight into the role of oxidation in defining the final composition and quality of black tea, emphasising the importance of controlled processing.

Advanced metabolomic tools, including random forest regression, k-means clustering, and enrichment analysis, provided a powerful framework for identifying chemical changes and pathway enrichments associated with tea processing. These techniques facilitated the detection of key metabolic shifts that would otherwise remain missed in targeted analyses, highlighting the dynamic transformations occurring during oxidation and roasting [12,13]. The integration of machine learning algorithms, such as random forest regression, allowed for the precise classification of tea processing stages based on metabolite profiles, demonstrating the effectiveness of predictive modelling in food metabolomics [14]. Enrichment analysis provided valuable insights into the functional implications of observed metabolic alterations, particularly in relation to flavonoid biosynthesis, amino acid metabolism, and carbohydrate pathways. These findings indicate the robustness of metabolomics in uncovering subtle variations in the chemical composition of tea, reinforcing its role as an essential tool for quality assessment, authentication, and process optimisation in tea production [24,25].

Furthermore, this research highlights the pivotal role of tea processing in determining its chemical and functional profiles. Refining processing techniques can enhance the retention of specific health-promoting compounds, offering opportunities for tailored production to meet increasing consumer demand for functional beverages [24]. Additionally, integrating metabolomic insights with sensory evaluation provides a holistic approach to tea quality assessment, bridging the gap between chemical diversity and consumer preferences and ultimately contributing to improved tea quality and product innovation [12,14].

## 5. Conclusions

This study provides new insight by integrating untargeted metabolomics with machine learning to putatively characterise, for the first time, the chemical transformations that occur during the processing of UK-grown tea. We identify previously unreported differences in catechin depletion between short and extended oxidation, alongside novel enrichment patterns across amino acid, carbohydrate, and flavonoid pathways. These findings offer a more detailed understanding of how processing duration modulates the metabolic landscape of both green and black tea. Together, these advancements extend the current knowledge of tea chemistry and highlight opportunities to refine processing strategies to enhance product quality and functional properties. Further work examining how environmental variables—including soil, climate, and microclimate—interact with processing conditions will help to deepen our understanding of factors shaping the chemical and sensory characteristics of UK-grown tea.

## Figures and Tables

**Table 1:** Functional and structural enrichment analysis was carried out on each cluster to identify putative chemical classes and biological functions associated with cluster trends, including those observed in green tea, black tea, and overnight processing.

	Cluster	KEGG Id	KEGG Name	*p*-Score
Green Tea	2	csin00290	Valine, leucine and isoleucine biosynthesis	0.000
	2	csin01210	2-Oxocarboxylic acid metabolism	0.000
	2	csin01230	Biosynthesis of amino acids	0.000
	2	csin01240	Biosynthesis of cofactors	0.000
	4	csin00941	Flavonoid biosynthesis	0.000
	6	csin00906	Carotenoid biosynthesis	0.000
	6	csin00941	Flavonoid biosynthesis	0.000
	6	csin01110	Biosynthesis of secondary metabolites	0.000
	6	csin04075	Plant hormone signal transduction	0.000
	7	csin00030	Pentose phosphate pathway	0.000
	7	csin00052	Galactose metabolism	0.000
	7	csin00280	Valine, leucine and isoleucine degradation	0.001
	7	csin00500	Starch and sucrose metabolism	0.000
	7	csin00511	Other glycan degradation	0.010
	7	csin00561	Glycerolipid metabolism	0.000
	7	csin00600	Sphingolipid metabolism	0.000
	7	csin00603	Glycosphingolipid biosynthesis	0.000
	7	csin00604	Glycosphingolipid biosynthesis	0.007
	7	csin00620	Pyruvate metabolism	0.041
	7	csin00640	Propanoate metabolism	0.000
	7	csin00945	Stilbenoid, diarylheptanoid and gingerol bios…	0.001
	7	csin02010	ABC transporters	0.000
	8	csin00052	Galactose metabolism	0.000
	8	csin00500	Starch and sucrose metabolism	0.000
	8	csin00600	Sphingolipid metabolism	0.000
	8	csin00603	Glycosphingolipid biosynthesis	0.000
	8	csin01110	Biosynthesis of secondary metabolites	0.000
	8	csin02010	ABC transporters	0.000
	9	csin00020	Citrate cycle (TCA cycle)	0.000
	9	csin00053	Ascorbate and aldarate metabolism	0.000
	9	csin00300	Lysine biosynthesis	0.000
	9	csin00480	Glutathione metabolism	0.000
	9	csin00620	Pyruvate metabolism	0.000
	9	csin00640	Propanoate metabolism	0.000
	9	csin00660	C5-Branched dibasic acid metabolism	0.027
	9	csin00941	Flavonoid biosynthesis	0.000
	9	csin01200	Carbon metabolism	0.000
	9	csin01210	2-Oxocarboxylic acid metabolism	0.000
	9	csin01230	Biosynthesis of amino acids	0.000
	11	csin00020	Citrate cycle (TCA cycle)	0.000
	11	csin00250	Alanine, aspartate and glutamate metabolism	0.036
	11	csin00290	Valine, leucine and isoleucine biosynthesis	0.043
	11	csin00660	C5-Branched dibasic acid metabolism	0.000
	11	csin00860	Porphyrin metabolism	0.000
	11	csin00920	Sulphur metabolism	0.000
	11	csin00941	Flavonoid biosynthesis	0.000
	11	csin00966	Glucosinolate biosynthesis	0.019
Black tea	3	csin00020	Citrate cycle (TCA cycle)	0.026
	3	csin00030	Pentose phosphate pathway	0.000
	3	csin00250	Alanine, aspartate and glutamate metabolism	0.001
	3	csin00350	Tyrosine metabolism	0.012
	3	csin00650	Butanoate metabolism	0.000
	3	csin00760	Nicotinate and nicotinamide metabolism	0.000
	3	csin01100	Metabolic pathways	0.017
	3	csin01200	Carbon metabolism	0.014
	5	csin00053	Ascorbate and aldarate metabolism	0.000
	5	csin00660	C5-Branched dibasic acid metabolism	0.000
	7	csin00053	Ascorbate and aldarate metabolism	0.002
	7	csin00130	Ubiquinone and other terpenoid-quinone biosyn…	0.000
	7	csin00350	Tyrosine metabolism	0.004
	7	csin00360	Phenylalanine metabolism	0.000
	7	csin00460	Cyanoamino acid metabolism	0.029
	7	csin00940	Phenylpropanoid biosynthesis	0.000
	7	csin00999	Biosynthesis of various plant secondary metab…	0.000
	8	csin00941	Flavonoid biosynthesis	0.000
	8	csin00944	Flavone and flavonol biosynthesis	0.000
	9	csin00944	Flavone and flavonol biosynthesis	0.000
	10	csin00220	Arginine biosynthesis	0.034
	10	csin00330	Arginine and proline metabolism	0.000
	10	csin00630	Glyoxylate and dicarboxylate metabolism	0.000
	10	csin00710	Carbon fixation in photosynthetic organisms	0.000
	12	csin00010	Glycolysis/Gluconeogenesis	0.000
	12	csin00051	Fructose and mannose metabolism	0.000
	12	csin00052	Galactose metabolism	0.000
	12	csin00510	N-Glycan biosynthesis	0.000
	12	csin00511	Other glycan degradation	0.000
	12	csin00513	Various types of N-glycan biosynthesis	0.000
	12	csin00531	Glycosaminoglycan degradation	0.000
	12	csin00562	Inositol phosphate metabolism	0.000
	12	csin00600	Sphingolipid metabolism	0.000
	12	csin00603	Glycosphingolipid biosynthesis	0.002
	12	csin00604	Glycosphingolipid biosynthesis	0.000
	12	csin02010	ABC transporters	0.000
	12	csin04070	Phosphatidylinositol signalling system	0.000
	12	csin04141	Protein processing in endoplasmic reticulum	0.000
	13	csin00040	Pentose and glucuronate interconversions	0.000
	13	csin00250	Alanine, aspartate and glutamate metabolism	0.000
	13	csin00460	Cyanoamino acid metabolism	0.000
	13	csin00470	D-Amino acid metabolism	0.000
	13	csin00543	Exopolysaccharide biosynthesis	0.000
	13	csin00908	Zeatin biosynthesis	0.000
	13	csin01232	Nucleotide metabolism	0.000
	13	csin04122	Sulphur relay system	0.000
	14	csin00220	Arginine biosynthesis	0.000
	14	csin00290	Valine, leucine and isoleucine biosynthesis	0.000
	14	csin00650	Butanoate metabolism	0.000
	14	csin01110	Biosynthesis of secondary metabolites	0.000
	14	csin01210	2-Oxocarboxylic acid metabolism	0.000
	14	csin01230	Biosynthesis of amino acids	0.000
	14	csin01240	Biosynthesis of cofactors	0.000
	15	csin00941	Flavonoid biosynthesis	0.000
Overnight	1	csin00020	Citrate cycle (TCA cycle)	0.000
	1	csin00053	Ascorbate and aldarate metabolism	0.000
	1	csin00480	Glutathione metabolism	0.000
	1	csin00660	C5-Branched dibasic acid metabolism	0.000
	1	csin00945	Stilbenoid, diarylheptanoid and gingerol bios…	0.000
	1	csin00999	Biosynthesis of various plant secondary metab…	0.001
	2	csin00020	Citrate cycle (TCA cycle)	0.000
	2	csin00250	Alanine, aspartate and glutamate metabolism	0.036
	2	csin00290	Valine, leucine and isoleucine biosynthesis	0.000
	2	csin00660	C5-Branched dibasic acid metabolism	0.000
	2	csin00860	Porphyrin metabolism	0.000
	2	csin00966	Glucosinolate biosynthesis	0.000
	3	csin00020	Citrate cycle (TCA cycle)	0.000
	3	csin00052	Galactose metabolism	0.000
	3	csin00053	Ascorbate and aldarate metabolism	0.000
	3	csin00500	Starch and sucrose metabolism	0.000
	3	csin00511	Other glycan degradation	0.001
	3	csin00531	Glycosaminoglycan degradation	0.003
	3	csin00561	Glycerolipid metabolism	0.031
	3	csin00600	Sphingolipid metabolism	0.000
	3	csin00603	Glycosphingolipid biosynthesis	0.000
	3	csin00604	Glycosphingolipid biosynthesis	0.000
	3	csin00620	Pyruvate metabolism	0.000
	3	csin01200	Carbon metabolism	0.035
	3	csin02010	ABC transporters	0.000
	5	csin00010	Glycolysis/Gluconeogenesis	0.000
	5	csin00051	Fructose and mannose metabolism	0.000
	5	csin00052	Galactose metabolism	0.000
	5	csin00510	N-Glycan biosynthesis	0.000
	5	csin00511	Other glycan degradation	0.000
	5	csin00513	Various types of N-glycan biosynthesis	0.000
	5	csin00562	Inositol phosphate metabolism	0.000
	5	csin02010	ABC transporters	0.000
	5	csin04070	Phosphatidylinositol signalling system	0.000
	5	csin04141	Protein processing in endoplasmic reticulum	0.000
	6	csin00010	Glycolysis/Gluconeogenesis	0.000
	6	csin00030	Pentose phosphate pathway	0.000
	6	csin00051	Fructose and mannose metabolism	0.031
	6	csin00280	Valine, leucine and isoleucine degradation	0.000
	6	csin00400	Phenylalanine, tyrosine and tryptophan biosyn…	0.048
	6	csin00410	beta-Alanine metabolism	0.005
	6	csin00520	Amino sugar and nucleotide sugar metabolism	0.000
	6	csin00620	Pyruvate metabolism	0.000
	6	csin00630	Glyoxylate and dicarboxylate metabolism	0.000
	6	csin00640	Propanoate metabolism	0.001
	6	csin00650	Butanoate metabolism	0.000
	6	csin00710	Carbon fixation in photosynthetic organisms	0.000
	6	csin00920	Sulphur metabolism	0.001
	6	csin01100	Metabolic pathways	0.002
	6	csin01200	Carbon metabolism	0.000
	6	csin01250	Biosynthesis of nucleotide sugars	0.040
	6	csin04148	Efferocytosis	0.000
	7	csin00941	Flavonoid biosynthesis	0.000
	8	csin00350	Tyrosine metabolism	0.000
	8	csin01100	Metabolic pathways	0.017
	9	csin00030	Pentose phosphate pathway	0.000
	9	csin00040	Pentose and glucuronate interconversions	0.000
	9	csin00220	Arginine biosynthesis	0.000
	9	csin00290	Valine, leucine and isoleucine biosynthesis	0.000
	9	csin00945	Stilbenoid, diarylheptanoid and gingerol bios…	0.000
	9	csin00960	Tropane, piperidine and pyridine alkaloid bio…	0.000
	11	csin01100	Metabolic pathways	0.017
	11	csin01110	Biosynthesis of secondary metabolites	0.000
	12	csin00260	Glycine, serine and threonine metabolism	0.000
	12	csin00330	Arginine and proline metabolism	0.004
	12	csin00410	beta-Alanine metabolism	0.000
	12	csin00630	Glyoxylate and dicarboxylate metabolism	0.000
	12	csin00770	Pantothenate and CoA biosynthesis	0.046
	13	csin01100	Metabolic pathways	0.017
	13	csin01110	Biosynthesis of secondary metabolites	0.000
	14	csin00030	Pentose phosphate pathway	0.000
	14	csin00130	Ubiquinone and other terpenoid-quinone biosyn…	0.000
	14	csin00290	Valine, leucine and isoleucine biosynthesis	0.000
	14	csin00310	Lysine degradation	0.012
	14	csin00350	Tyrosine metabolism	0.005
	14	csin00360	Phenylalanine metabolism	0.000
	14	csin00400	Phenylalanine, tyrosine and tryptophan biosyn…	0.000
	14	csin00562	Inositol phosphate metabolism	0.000
	14	csin00650	Butanoate metabolism	0.006
	14	csin00750	Vitamin B6 metabolism	0.000
	14	csin00940	Phenylpropanoid biosynthesis	0.000
	14	csin00944	Flavone and flavonol biosynthesis	0.000
	14	csin00950	Isoquinoline alkaloid biosynthesis	0.000
	14	csin01210	2-Oxocarboxylic acid metabolism	0.016
	14	csin01230	Biosynthesis of amino acids	0.000
	14	csin04075	Plant hormone signal transduction	0.007
	15	csin00020	Citrate cycle (TCA cycle)	0.000
	15	csin00053	Ascorbate and aldarate metabolism	0.000
	15	csin00300	Lysine biosynthesis	0.000
	15	csin00480	Glutathione metabolism	0.000
	15	csin00620	Pyruvate metabolism	0.000
	15	csin00640	Propanoate metabolism	0.000
	15	csin00660	C5-Branched dibasic acid metabolism	0.000
	15	csin00941	Flavonoid biosynthesis	0.000
	15	csin01200	Carbon metabolism	0.004
	15	csin01210	2-Oxocarboxylic acid metabolism	0.003
	15	csin01230	Biosynthesis of amino acids	0.000

**Table 2:** Functional and structural enrichment analysis was performed on the *m*/*z* features that were found to be increased or decreased in the overnight treatments compared to the 4 h oxidation treatments and after roasting. The putatively identified, significantly enriched metabolic pathways are shown.

Comparison	Trend	KEGG ID	KEGG Name	*p*-Score
Oxidation (4 h)~Oxidation (overnight)	decreased	csin00052	Galactose metabolism	0.000001
	decreased	csin00350	Tyrosine metabolism	0.00094
	decreased	csin00360	Phenylalanine metabolism	0.00158
	decreased	csin00500	Starch and sucrose metabolism	0.000001
	decreased	csin00600	Sphingolipid metabolism	0.000001
	decreased	csin00603	Glycosphingolipid biosynthesis	0.000001
	decreased	csin00940	Phenylpropanoid biosynthesis	0.0000405
	decreased	csin00941	Flavonoid biosynthesis	0.000001
	decreased	csin00944	Flavone and flavonol biosynthesis	0.000941
	decreased	csin00999	Biosynthesis of various plant secondary metabolite.	0.000001
	decreased	csin02010	ABC transporters	0.000001
	increased	csin00040	Pentose and glucuronate interconversions	0.000001
	increased	csin00051	Fructose and mannose metabolism	0.000001
	increased	csin00053	Ascorbate and aldarate metabolism	0.000001
	increased	csin00290	Valine, leucine and isoleucine biosynthesis	0.000001
	increased	csin00310	Lysine degradation	0.000001
	increased	csin00562	Inositol phosphate metabolism	0.000001
	increased	csin00630	Glyoxylate and dicarboxylate metabolism	0.0000296
	increased	csin00660	C5-Branched dibasic acid metabolism	0.000001
	increased	csin00750	Vitamin B6 metabolism	0.00000455
	increased	csin00760	Nicotinate and nicotinamide metabolism	0.000001
	increased	csin00770	Pantothenate and CoA biosynthesis	0.000001
	increased	csin00906	Carotenoid biosynthesis	0.000001
	increased	csin00945	Stilbenoid, diarylheptanoid and gingerol biosynthe…	0.0000448
	increased	csin01230	Biosynthesis of amino acids	0.0000232
Roasting (4 h)~Roasting (overnight)	decreased	csin00020	Citrate cycle (TCA cycle)	0.000001
	decreased	csin00052	Galactose metabolism	0.0000184
	decreased	csin00053	Ascorbate and aldarate metabolism	0.000001
	decreased	csin00062	Fatty acid elongation	0.00129
	decreased	csin00500	Starch and sucrose metabolism	0.000001
	decreased	csin00600	Sphingolipid metabolism	0.0000352
	decreased	csin00603	Glycosphingolipid biosynthesis	0.000001
	decreased	csin00620	Pyruvate metabolism	0.0228
	decreased	csin00941	Flavonoid biosynthesis	0.000001
	decreased	csin00944	Flavone and flavonol biosynthesis	0.0183
	decreased	csin00999	Biosynthesis of various plant secondary metabolite.	0.000001
	decreased	csin02010	ABC transporters	0.00000268
	increased	csin00010	Glycolysis/Gluconeogenesis	0.00000109
	increased	csin00040	Pentose and glucuronate interconversions	0.000001
	increased	csin00051	Fructose and mannose metabolism	0.000001
	increased	csin00053	Ascorbate and aldarate metabolism	0.000001
	increased	csin00290	Valine, leucine and isoleucine biosynthesis	0.000001
	increased	csin00310	Lysine degradation	0.000001
	increased	csin00562	Inositol phosphate metabolism	0.000001
	increased	csin00630	Glyoxylate and dicarboxylate metabolism	0.000001
	increased	csin00650	Butanoate metabolism	0.000001
	increased	csin00760	Nicotinate and nicotinamide metabolism	0.000001
	increased	csin02010	ABC transporters	0.000001

## Data Availability

The metabolomics and metadata reported in this paper are available on request.

## Supplementary Materials

The following supporting information can be downloaded at: https://www.mdpi.com/article/10.3390/metabo16010084/s1, Supplementary S1.

## Abbreviations

FIE-MS	Flow Infusion Electrospray Ionisation Mass Spectrometry
EC	(-)-epicatechin
EGC	(-)-epigallocatechin
ECG	(-)-epicatechin-3-gallate
EGCG	(-)-epigallocatechin-3-gallate
HPLC	High-performance liquid chromatography
MS	Mass Spectrometry
